# Numb Chin Syndrome Leading to a Diagnosis of Salivary Ductal Adenocarcinoma: A Case Report and Review of the Literature

**DOI:** 10.3389/fneur.2017.00343

**Published:** 2017-07-25

**Authors:** Lei Wu, Yifan Zheng, Zhou Zhou, Yanmei Liu, Weixi Zhang, Qi Wu

**Affiliations:** ^1^Department of Neurology, The First Affiliated Hospital, Sun Yat-sen University, Guangzhou, China

**Keywords:** numb chin syndrome, mental nerve neuropathy, metastatic tumor of mandible, malignant, salivary carcinoma, salivary ductal adenocarcinoma

## Abstract

Numb chin syndrome (NCS) refers to a rare sensory neuropathy characterized by numbness of the chin within the distribution of the mental or inferior alveolar nerve. Although NCS is usually caused by a benign process, it should not be underestimated and a thorough diagnostic evaluation for a new or known progressive malignancy should always be performed. Here, we report a case of salivary ductal adenocarcinoma that mimicked a pulpitis and periodontitis in its early presentation accompanied by numbness of chin. The course and diagnosis of this case are discussed, and a brief review of the literature is presented. It is hoped for clinicians to keep the malignant possibility of NCS in mind and take a thorough examination.

## Introduction

Numb chin syndrome (NCS), often synonymously named as “mental nerve neuropathy,” is a sensory neuropathy characterized by numbness (hypoesthesia, paresthesia, dysesthesia, and anesthesia) or, very rarely, pain of the chin and lower lip within the mental or inferior alveolar nerve distribution. The causes of NCS can be diverse. Most cases are caused by odontogenic diseases such as trauma, dental extraction, dentoalveolar abscess, and osteomyelitis (1, 2). However, this innocuous complaint is sometimes considered as a “red flag” symptom of an incipient malignancy or an indication of the spread of an established tumor.

Compared to intraoral mucosal malignancies, NCS does relate more to metastatic tumors. Intraoral mucosal malignancies, such as squamous cell carcinoma of the oral mucosa or lip or cancer of the small salivary glands, are usually associated with typical mucosal signs, for example, ulceration with raised margins, lumps with abnormal vessels, or abnormal swellings. A provisional suspicion of malignancy usually results from clinical presentation in most cases. However, NCS, where mucosal findings are not present, is usually a single oral maxillofacial presentation induced by metastasis of remote malignancies and therefore requires a thorough examination to make prompt and accurate diagnosis (3–5).

In this paper, aimed to highlight that NCS might lead to severe conditions and to show how the diagnosis was made, we reported a case with an initial character of NCS that was finally confirmed as a mandible malignancy originating from salivary duct adenocarcinoma and reviewed the causes, the possible mechanism, the diagnostic approaches, and differential diagnosis of NCS.

## Case Report

A 64-year-old man with a persistent pain in his lower front teeth, which made him dare not to chew for a few days, was diagnosed as pulpitis by his dentist. After a root canal treatment of teeth #42 to #32 [International Standards Organization Designation System (ISO System)], his toothache relieved a little. However, numbness on the left side of his chin occurred and progressed gradually to involve his entire chin and low lip. A month later, a throbbing pain with no trigger point attacked his chin, appearing several times every day, and lasting for hours. Chewing or touch could increase the pain intensity. The pain became so acute to affect his sleep. It was diagnosed as periodontitis and treated with antibiotics and analgesics, but the numbness and pain of the chin got worse. Magnetic resonance imaging (MRI) of the trigeminal nerves revealed a small vessel riding across the left trigeminal nerve and multiple patchy abnormal signals in pons, bilateral frontal and parietal lobes. To determine what exactly caused the trouble, he was then admitted to the neurology ward.

He had no other symptoms such as headache, visual disorders, difficulties in swallowing, and speech, limb weakness, or numbness. No weight lost in the past months. His past medical history was uncontrolled hypertension. He smoked 20 cigarettes a day for 40 years and only drank a little alcohol occasionally.

**Figure 1:**
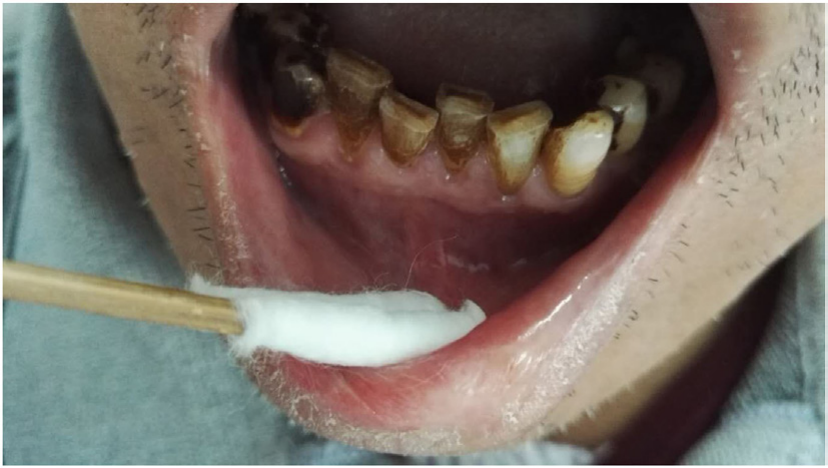
Clinical examination showing his black teeth with enhancing accumulations of plaque calculus. No abnormal protuberance in gingival cheek groove.

On physical examination, his teeth were black with enhancing accumulations of plaque calculus (Figure 1). The oral mucosa appeared normal. His general physical examination was unremarkable with no cervical lymphadenopathy. Anesthesia was present over his chin and lower lip bilaterally while the sensation over the rest of his face was normal. His corneal reflexes and his bite force were normal. Examination of other cranial nerves and limbs including motion, sensation, and reflex was normal.

Hemanalysis showed normal in full blood count, erythrocyte sedimentation rate, plasma glucose, urea and electrolytes, serum C reactive protein, creatinine, liver function, and immune indices. Syphilis antibody and tumor markers were all negative.

According to the symptoms, signs, and MRI result, a diagnosis of NCS due to the mental nerve inflammation induced by periodontitis was made. It was differentiated from the trigeminal neuralgia and central nervous system demyelinating disease such as multiple sclerosis. The MRI showed a vessel riding across but not compressing the trigeminal nerve, and pain and numbness affected the entire chin with no trigger point. These were inconsistent with typical trigeminal neuralgia. Central nervous system demyelinating diseases were also excluded by lack of supports such as other symptoms and signs of nervous system, normal cerebrospinal fluid, negative AQP-4 antibody and oligoclonal band, and normal evoked potentials (somatosensory, brainstem auditory, and visual). The character of the abnormal signals in the outpatient MRI was defined as ischemic lesions by a brain MRA + DWI + SWI performed after admission, which was also not thought to be responsible for the symptoms.

**Figure 2:**
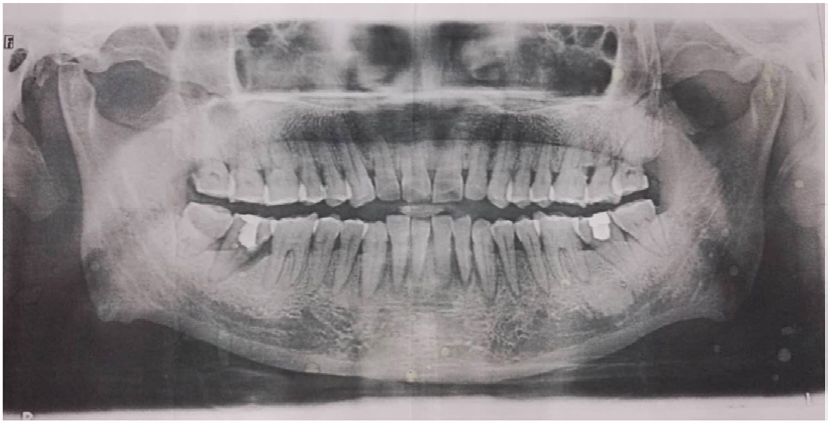
The panoramic radiography of the jaw was normal.

After the diagnosis was made, the patient was treated with pregabalin, prednisone, and vitamins. The pain relieved a little and the sensation over his chin and lower lip recovered to some degree, especially on the right side. Unfortunately, a week later, the symptoms became worse again and progressed gradually to a degree that painkiller was needed to help him fall asleep. He was sent to the dentist and found several lower front teeth loose and gingival sulcus swelling. The panoramic radiography of the jaw was normal (Figure 2). It seemed to confirm the previous diagnosis.

**Figure 3:**
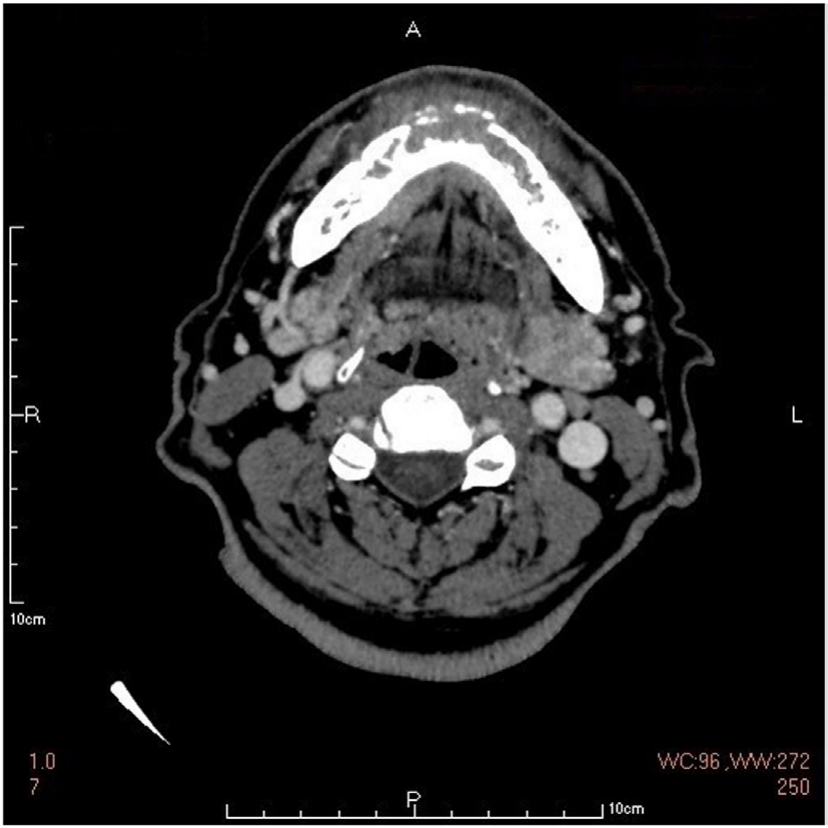
The mandibular computerized tomography scan showing destruction in the body of mandibular bone and a mass in the surrounding soft tissue.

**Figure 4:**
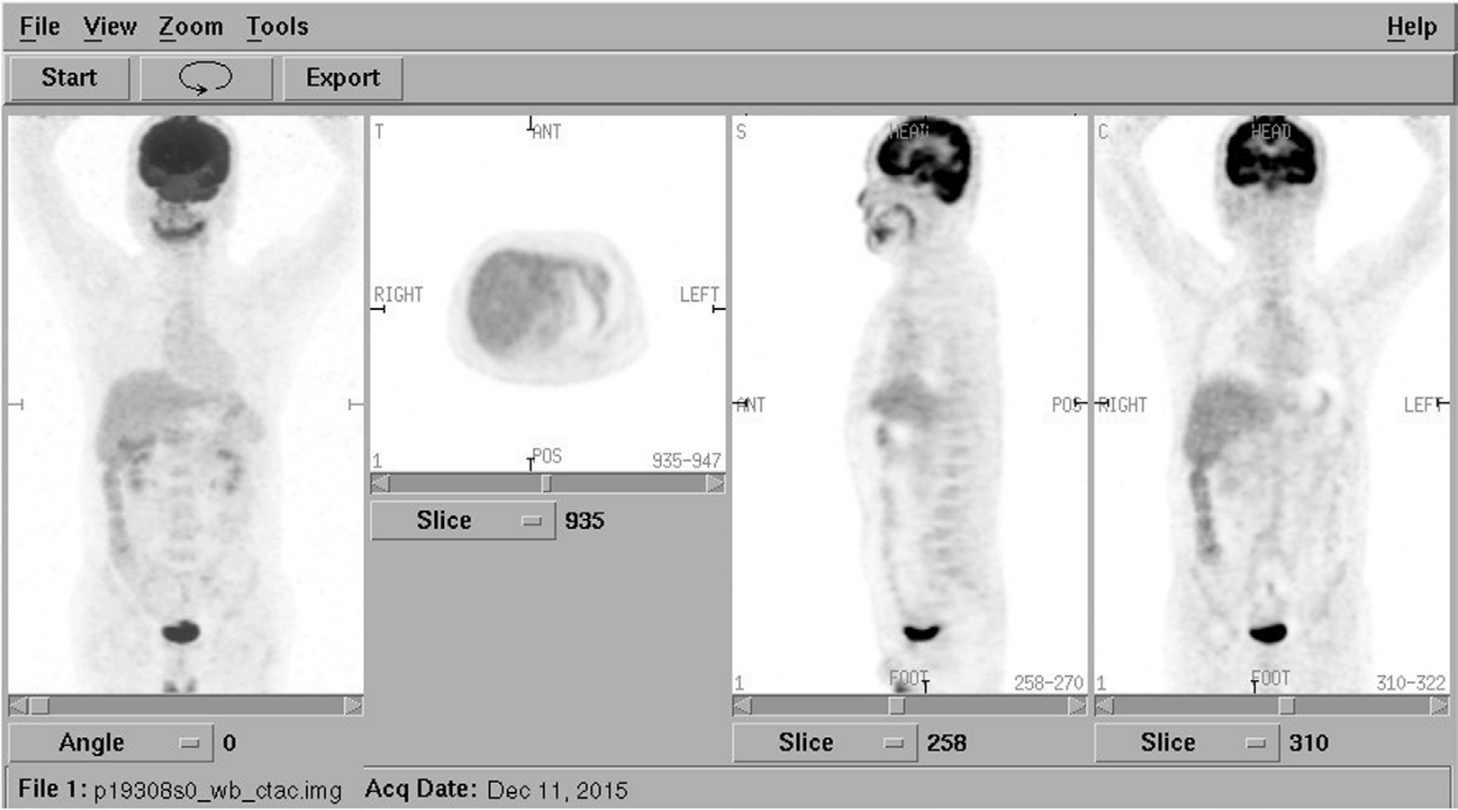
Positron emission tomography combined with computed tomography from the cerebellum to the upper thighs showing increased uptake in the mandibular bone body especially in the left mandible. No abnormal uptake in distant sites.

However, considering the poor response to the treatment and the unusual looseness involving several of the lower anterior teeth, a mandibular computerized tomography (CT) scan was performed to exclude an underlying malignancy that was easily ignored in NCS. It revealed destruction in the body of mandibular bone and a mass in the surrounding soft tissue, which was considered as a possible malignancy, most likely to be the gingival cancer (Figure 3). A positron emission tomography combined with computed tomography (PET-CT) from the cerebellum to the upper thighs showed increased uptake in the mandibular bone body especially in the left mandible (Figure 4).

The patient was then admitted to dental ward for surgery. On dental examination, a bony distention was palpable on the right mandibular symphysis, boundary clear, with mild tenderness. The sensation over the chin and lower lip was decreased. No limitation of mouth opening. Teeth #44 to #34 (ISO System) loose in II–III degree. None of the gum, bilateral parotid, or submandibular gland conduit mouth was swollen. No enlarged lymph node was palpable.

**Figure 5:**
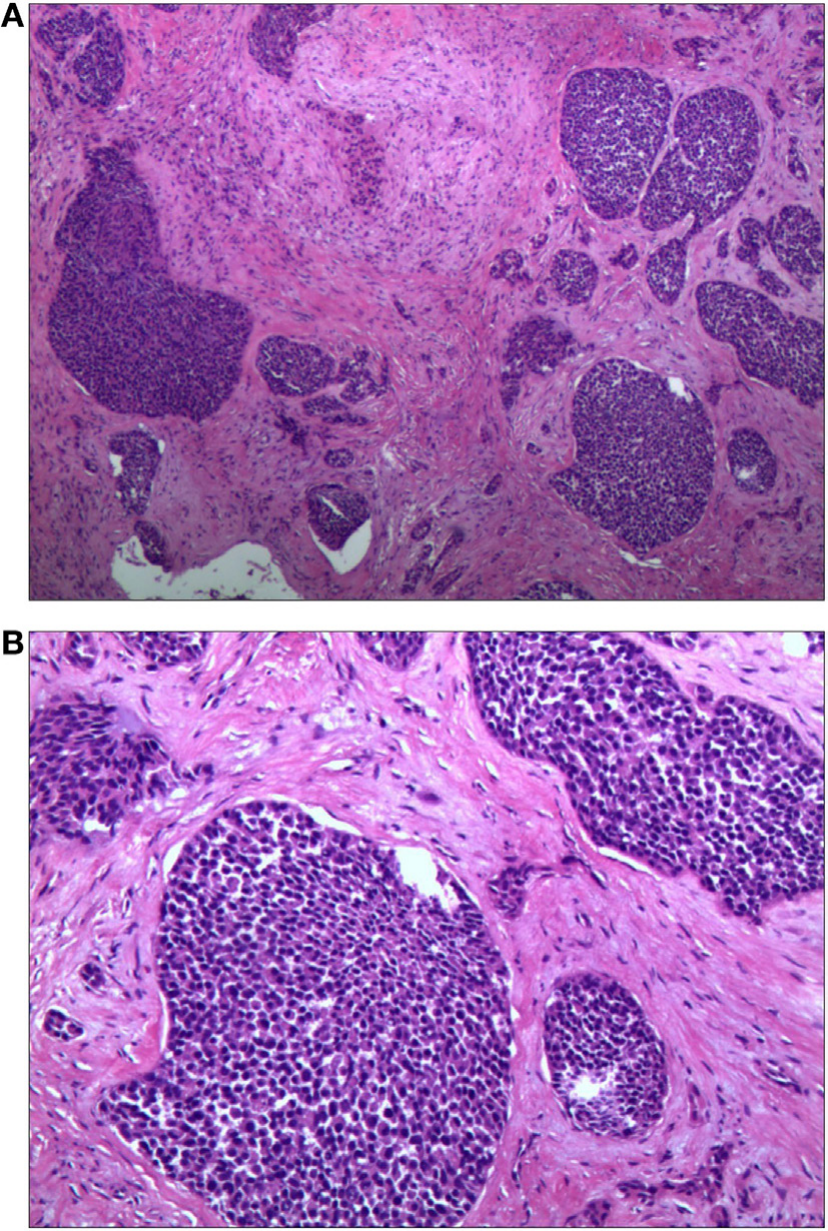
Histopathological examination of the biopsy specimen showing infiltration of carcinoma cells with nest-like distribution. The carcinoma cells, round and oval in shape and most in mitosis, were abundant of cytoplasm [H&E **(A)** 40× and **(B)** 100×].

He underwent a maxillofacial surgery and the tumor was resected. The histopathological examination showed infiltration of carcinoma cells with nest-like distribution in the fibrous tissue and bone (Figure 5). The carcinoma cells, round and oval in shape and most in mitosis, were abundant of cytoplasm. The pathomorphological features revealed an epithelial malignant tumor, which was considered as a ductal adenocarcinoma derived from salivary gland with potentially low differentiation. Both of the two inferior alveolar nerves were invaded by tumor and metastases were discovered in the right submandibular lymph nodes.

The patient then received chemoradiotherapy; however, he still developed widespread metastasis and died after 1 year from the onset of his chin numbness.

## Discussion

Numb chin syndrome is an infrequently recognized neurological disorder presenting with numbness over the lower lip and chin. It has not been reported in China yet. NCS is caused by the lesion of the mental nerve which is one of the terminal branches of the mandibular division of the trigeminal nerve (6). Although NCS is unilateral in most cases, it may be bilateral in a few cases (10%) which may occur simultaneously or subsequently as seen in our case (7). Because the mental nerve has no motor fibers, motor function of the lower face is intact in patients with NCS.

Anatomically, the mandibular division of the trigeminal nerve, after exiting the skull base through the foramen ovale, branches into the inferior alveolar nerve passing through the mandible canal, and finally exits at the mental foramen as the mental nerve. The mental nerve supplies the sensation of the chin and lower lip (8, 9). Any pathological process affecting the mental nerve and the mandibular nerve may lead to paresthesia of the chin, lower lip, and gingival mucosa.

Numb chin syndrome is usually thought as an isolated neurological lesion but not as part of an extensive neurological disorder such as a part of a stroke or demyelinating process. Some neurological diseases, such as multiple sclerosis, Lyme disease, or strokes (10, 11) and diabetes mellitus (12), also may lead to NCS in a broader concept which usually companied with some other damages of the nerve system. When a patient with NCS presents to a neurologist initially, a complete neurological examination should be done to recognize the trigeminal neuropathy and the presence of other neurological deficits accompanied with paresis, ataxia, or impairment of further cranial nerves.

Numb chin syndrome is thought to be mostly caused by odontogenic conditions such as infection, trauma, and dental procedures (1, 2). However, this innocuous symptom familiar to anyone having had local dental anesthesia may betray a more alarming and underlining disease. Although rare, it may be the first symptom of an underlying malignancy (6). In this case, the numbness preceded by the feeling of toothache was considered to be caused by dental problem at first. Because of the poor reaction to the root canal treatment and some medicines such as pregabalin, prednisone, and vitamins, examinations including CT scan and PET-CT were performed and revealed a malignancy in the mandibular bone body, which was pathologically confirmed as a ductal adenocarcinoma derived from salivary gland with potentially low differentiation.

According to reports, the causes of NCS were invasive dental procedures (63%), inflammation (15%), and malignancy (22%) (13). NCS generally appears when the tumor recurs or metastasizes, 4 years after the first malignancy diagnosis on average, and it can also precede the diagnosis of cancer (47%) (14). The most common primary cancers are breast cancer, lung cancer, lymphoma, and cancers in thyroid, prostate, and colon, although melanoma, myeloma, sarcoma, and cancers in ovary, testis, salivary glands, lip, and gut have also been reported. Breast cancer and lymphoma account for most cases of NCS in adults, while acute lymphoblastic leukemia is a significant cause in children (6, 7). Histologically, adenocarcinoma originating from different tissues is the most frequent subtype reported in the literature (70%) (15). However, ductal adenocarcinoma originating from salivary gland, the pathological subtype of this case, has not been reported yet in NCS. Although other salivary gland carcinomas also are known for their tendency for perineural tumor invasion, such as adenoid cystic carcinoma (ACC), which has a putative intercalated duct origin. The difference is that ACC is histologically composed of mainly myoepithelial cells, but the immunohistochemical markers for myoepithelial cells such as Calponin and P63 were negative in our case. What’s more, growth of ACC is slower, with the 5-year survival rates are very favorable at 70–90% (16).

The mechanism by which NCS occurs in connection with neoplasm is still unknown, although several hypotheses have been raised. A number of pathological mechanisms may account for the neuropathy: the compression or infiltration to the mental or the inferior alveolar nerve by metastasis, intracranial involvement particularly near the Gasserian ganglion by metastases at the skull base arising from lymphatic orhematogenous spread (17–20).

As NCS can be caused by diverse pathologies either benign or malignant, it is necessary to consider it as a serious problem that requires a thorough medical history, clinical examination, blood and cerebrospinal fluid analysis, and imaging to make a certain diagnosis.

As far as imaging, panoramic jaw radiograph, CT, MRI, or Gadolinium-enhanced MRI of the brain and even PET-CT may be needed in diagnosis of NCS. The panoramic radiography is usually the first imaging study used in patients with NCS, but it may fail to detect soft tissue tumors and those inside the nerve canal as that in this case (21). Bone invasion may initially occur without radiographic changes because of infiltration through marrow spaces. CT and MRI are more helpful than standard X-rays for further diagnosis of NCS. CT scan of the brain and mandible can show bony lesions or damage of skull base while MRI scan (particularly with gadolinium enhancement) can detect nerve involvement, intracranial disease like trigeminal ganglion enlargement and leptomeningeal invasion (22). MRI is often used to evaluate the trigeminal nerve branches and to exclude other diseases such as stroke and multiple sclerosis. However, a classical brain MRI protocol may sometimes not extend inferiorly enough to view the mental foramen and may therefore miss a focal mass or osseous lesion (23). In addition, the diagnostic process may require thoracic or abdominal radiographs, sonography, and, if needed, abdominal CT scans and MRI, PET-CT scans to look for primary neoplasm and its metastatic sites (6, 24). The patient in this case was once suspected as trigeminal neuralgia because the MRI showed a vessel riding across the trigeminal nerve. The soft tissue mass in the mandibula was not found until the mandibular CT was taken. It was confirmed as a metastasis by PET-CT and a ductal adenocarcinoma pathologically.

The treatment and prognosis of NCS are different according to various etiologies. Patients with NCS caused by dental diseases may recover after the local conditions have improved, while those caused by malignancy are usually treated by analgesic and antitumor therapy with little effect and poor prognosis. The mean survival in many cases is only 6 months or less (7). In this case, the patient received an operation accompanied by chemoradiotherapy but died after 1 year from the onset of his chin numbness. His survival time was longer than the mean survival reported, which may be benefited by receiving prompt diagnosis and treatment before severe distant metastasis.

## Conclusion

Numb chin syndrome may often be ignored by patients and clinicians because the symptom is not so serious to affect patients’ daily activities. However, NCS could sometimes be a clue of metastatic malignancies. Recognizing the potential clinical significance is the most important step in the diagnosis of NCS. NCS patients, with a history of cancer or not responding to conventional management for a prolonged time span, should undergo specific and thorough investigations to rule out a malignancy. We recommend that all medical practitioners and dentists should be aware of NCS and its possible implication to malignancies. For a NCS without any obvious odontogenic causes, examinations should be done as soon as possible to confirm or exclude metastatic disease. In addition, the limitations of orthopantomogram or a normal brain MRI on the detection of underlying mandible diseases should be recognized.

## Ethics Statement

No investigation or intervention was performed outside routine clinical care for this patient. As this is a case report, without experimental intervention into routine care, no formal research ethics approval is required. Written, fully informed consent was given and recorded from the patient in clinical process. Since the patient had already been dead when we summarized this case, his son wrote a fully informed consent for publication.

## Author Contributions

LW, ZZ, YL, WZ, and QW were involved in the work-up of the patient, planning and conducting investigations, and providing clinical care. They reviewed and revised the manuscript and approved the final manuscript as submitted. LW, YZ, WZ, and QW planned the case report, drafted the initial manuscript, reviewed and revised the manuscript, and approved the final manuscript as submitted.

## Conflict of Interest Statement

The authors declare that the research was conducted in the absence of any commercial or financial relationships that could be construed as a potential conflict of interest. The reviewer, JL, and handling editor declared their shared affiliation, and the handling editor states that the process nevertheless met the standards of a fair and objective review.
